# Supplementary material for: Anaplerotic flux into the Calvin–Benson cycle: hydrogen isotope evidence for in vivo occurrence in C3 metabolism
Source: New Phytol. 2022 Feb 2;234(2):405–11. doi: 10.1111/nph.17957 (PMC9305100; doi:10.1111/nph.17957)
Supplement: Supplementary file 1 — Notes S1 Leaf gas exchange measurements. Notes S2 Estimation of intercellular CO2 concentrations during growth chamber experiments. Notes S3 Dilution of isotope signals by remnant starch. Notes S4 Deuterium fractionation by glucose‐6‐phosphate dehydrogenase. Notes S5 Deuterium fractionation by phosphoglucose isomerase. Please note: Wiley Blackwell are not responsible for the content or functionality of any Supporting Information supplied by the authors. Any queries (other than missing material) should be directed to the New Phytologist Central Office. [file NPH-234-405-s001.pdf]

Anaplerotic flux into the Calvin-Benson cycle. Hydrogen isotope evidence for *in vivo* occurrence in C_3 metabolism – Supporting information

Thomas Wieloch, Angela Augusti, Jürgen Schleucher (Accepted: 22 December 2021)

Notes S1. Leaf gas exchange measurements

Gas exchange measurements were performed with a two-channel fast-response system described previously (Laisk & Edwards, 1997). A sandwich-type round chamber enclosed a leaf area of 8.04 cm^2. Gas flow was maintained at 0.5 mmol s^{-1}. Measurements were performed at CO_2 concentrations similar to those applied in the growth chamber over two days (C_a=173 ± 0.1SE, 269 ± 0.1SE, 433 ± 0.1SE, 675 ± 0.2SE, 1431 ± 0.4SE ppm; n=8). To maintain temperatures of 22 ± 0.5 °C, the upper leaf side was attached to a thermostated glass window by starch gel. Light was provided by a Schott KL 1500 lamp (Walz Heinz GmbH, Effeltrich, Germany) and measured by a LI-185 quantum sensor (LiCor, Lincoln, USA). Measurements were performed at 300 µmol photons m^{-2} s^{-1} yielding C_i=136 ± 1SE, 202 ± 4SE, 304 ± 8SE, 516 ± 14SE, 1282 ± 16SE ppm.

Notes S2. Estimation of intercellular CO_2 concentrations during growth chamber experiments

Figure S1 shows C_a and C_i data obtained by gas exchange analysis (black circles). Their relationship is well described by a quadratic equation (dotted line, R^2=0.999, p<0.001, n=5). Based on this equation, we estimated C_i values of 140, 206, 328, 531, and 1365 ppm for growth chamber treatments of C_a=180, 280, 450, 700, and 1500 ppm (red circles).

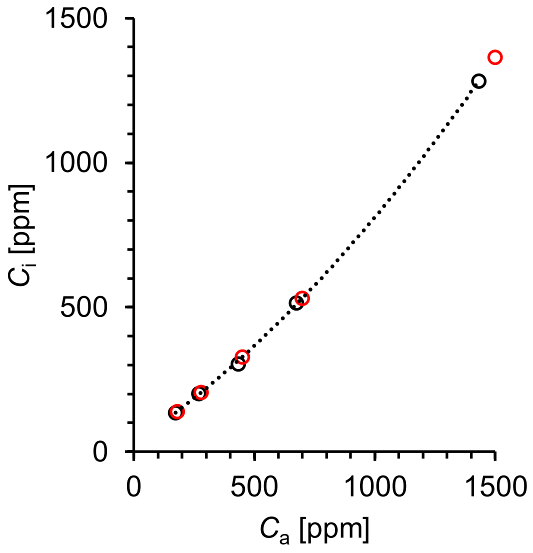

Figure S1 Ambient and intercellular CO_2 concentrations in *Helianthus annuus* leaves (C_a, and C_i, respectively). Black circles: measured values obtained by gas exchange analysis. Black dotted line: quadratic relationship between C_a and C_i measurements (R^2=0.999, p<0.001, n=5). Red circles: C_i values during growth chamber experiments estimated based on the quadratic equation.

Notes S3. Dilution of isotope signals by remnant starch

The plants studied here were grown at a CO_2 concentration of 450 ppm over 7 to 8 weeks. H^1 and H^2 of starch glucose synthesised under these conditions have δD values of -247‰ and -557‰, respectively (Fig. 3). To drain the starch reserves and avoid dilution by 450 ppm isotope signals, all plants were kept in darkness for 24 hours after transfer to growth chambers. Reportedly, this treatment led to a starch reduction from about 8 to almost 0 μmol glucose g^{-1} FW in *Arabidopsis thaliana* (Smith & Stitt, 2007). Similarly, we found a starch remnant of <0.6 μmol glucose g^{-1} FW in our samples. After two days of acclimation to 1500 ppm, we found a starch content of >171 μmol glucose g^{-1} FW. Thus, at 1500 ppm CO_2, dilution of isotope signals due to remnant (450 ppm) starch is negligible (0.6/171=4‰).

At 180 ppm, net CO_2 assimilation was lower than at 1500 ppm (5.1 vs. 17.3 μmol m^{-2} s^{-1}). Assuming equal relative carbon partitioning under both conditions, dilution of isotope signals in starch synthesised at 180 ppm is small (0.6/(171*5.1/17.3)=1.2%). H^1 and H^2 of starch glucose synthesised at 180 ppm have apparent δD values of -37‰ and -246‰, respectively (Fig. 3). Accounting for a 1.2% dilution by remnant starch, we estimate actual δD values of -34.4‰ and -242.2‰, respectively. However, sucrose-to-starch carbon partitioning ratios increase as ambient CO_2 concentrations decrease (Sharkey et al. 1985). Thus, at 180 ppm CO_2, the relative contribution of remnant (450 ppm) starch may be larger, i.e., actual δD values and anaplerotic flux may be somewhat higher than estimated.

Notes S4. Deuterium fractionation by glucose-6-phosphate dehydrogenase

Modelling of fractionations by glucose-6-phosphate dehydrogenase, G6PD, assumed an open system at steady state and followed published basic procedures (Hayes, 2002). Incoming glucose 6-phosphate, G6P, has two fates, starch biosynthesis or anaplerotic reinjection into the Calvin-Benson cycle via G6PD. D fractionation between the reaction product, 6-phosphogluconolactone (6PGL), and remaining educt, G6P', is given as

$$R_{\mathrm{6PGL}} = \frac{1}{\alpha} R_{\mathrm{G6P'}} \qquad \text{(S1)}$$

where R denotes D/H ratios, and α denotes D isotope effects of G6PD (α_D=2.97) (Hermes *et al.*, 1982). Isotope mass balance of the system is given as

$$F_{\mathrm{G6P}} = fF_{\mathrm{6PGL}} + (1-f)F_{\mathrm{G6P'}} \quad \text{(S2)}$$

where F denotes fractional abundances (D/(H+D)), and f denotes the 6PGL commitment (f was varied between 0 and 1). F and R relate to each other as

$$F = \frac{R}{1+R} \quad \text{(S3)}$$

Substituting F for R in equation S2 yields

$$\frac{R_{\mathrm{G6P}}}{1+R_{\mathrm{G6P}}} = f\frac{R_{\mathrm{6PGL}}}{1+R_{\mathrm{6PGL}}} + (1-f)\frac{R_{\mathrm{G6P'}}}{1+R_{\mathrm{G6P'}}} \quad \text{(S4)}$$

Here, we substitute the unknown D/H ratio of incoming G6P, R_{G6P}, by the known ratio of Vienna Standard Mean Ocean Water, R_{VSMOW} ($155.76 \cdot 10^{-6}$) (Hagemann *et al.*, 1970). Consequently, fractionations expressed on the δ-scale (Eq. S7) develop from zero. Substituting R_{6PGL} in equation S4 based on equation S1 then gives

$$\frac{R_{\mathrm{VSMOW}}}{1+R_{\mathrm{VSMOW}}} = f\frac{R_{\mathrm{G6P'}}/\alpha}{1+R_{\mathrm{G6P'}}/\alpha} + (1-f)\frac{R_{\mathrm{G6P'}}}{1+R_{\mathrm{G6P'}}} \quad \text{(S5)}$$

Solving equation S5 for $R_{\mathrm{G6P'}}$ gives

$$c = \frac{-ad - a + b + d - bd \pm Z}{2(a-1)} \quad \text{(S6)}$$

with

$$Z = \sqrt{a^2d^2 - 2ad^2 + b^2d^2 + 2abd^2 - 2bd^2 + d^2 - 2a^2d + 2ad - 2b^2d + 2bd + a^2 + b^2 - 2ab}$$

where a to d denote $R_{\mathrm{VSMOW}}/(1+R_{\mathrm{VSMOW}})$, f, $R_{\mathrm{G6P'}}$, and α, respectively. While there are two mathematically correct solutions ($\pm Z$), only one of them returns plausible fractionation values ($-Z$). Modelling results were expressed in terms of R_{VSMOW} as

$$\delta D_{G6P'} = \frac{R_{G6P'}}{R_{VSMOW}} - 1 \quad \text{(S7)}$$

Notes S5. Deuterium fractionation by phosphoglucose isomerase

In the F6P to G6P direction, spinach PGI has a tritium isotope effects, α_T, of 3 (Noltmann, 1972). The corresponding deuterium isotope effect, α_D, can be estimated from α_T as

$$\alpha_D = k_H/k_D = \sqrt[1.44]{\alpha_T} \quad \text{(S8)}$$

where k_H and k_D denote the reaction rates of the protium and deuterium isotopologues of F6P, respectively (Melander & Saunders, 1980). Based on this relationship, we estimate an α_D of 2.14 which may cause D depletions at G6P H^2 of about 534‰ ($1/\alpha_D$-1).

For rabbit muscle PGI, Rose and O'Connell (1961) measured an α_D of 2.22 in the F6P to G6P direction (Table S1) which is similar to the α_D estimated for spinach PGI. In the G6P to F6P direction, these authors measured an α_D of 2. From these values, we calculated an equilibrium isotope effect, EIE_D, of 1.11 in G6P. The kinetic isotope of effect (F6P to G6P direction, α_D=2.22) may cause D depletions at G6P H^2 of about 550‰ while the equilibrium isotope effect (EIE_D=1.11) may cause D depletions of about 99‰ ($1/EIE_D$-1). Hence, with a shift from kinetic to equilibrium conditions, the D abundance at G6P H^2 may increase by about 451‰.

Table S1 *In vitro* D isotope effects of PGI (Rose & O'Connell, 1961).

F6P		G6P
	→	2.22
<u>0.9</u>	↔	<u>1.11</u>
2	←	

→ kinetic isotope effect, ↔ equilibrium isotope effect. Calculated values underlined.

Note, our discussion implicitly assumes that all hydrogen from F6P H^{1R} is transferred to G6P H^2. However, at 22 °C, spinach PGI transfers only 70% of the hydrogen while the rest undergoes exchange with the medium (F6P to G6P direction) (Noltmann, 1972). To our knowledge, isotope effects of hydrogen loss to the medium and hydrogen uptake from the medium are unknow as is the hydrogen isotope composition of the medium. Furthermore, hydrogen exchange rates are temperature dependent and differ between the forward and

backward reaction of PGI (Rose & O'Connell, 1961; Noltmann, 1972). Thus, isotope fractionation by hydrogen exchange requires further attention.

References

Hagemann R, Nief G, Roth E. **1970**. Absolute isotopic scale for deuterium analysis of natural waters. Absolute D/H ratio for SMOW. *Tellus* **22**: 712–715.

Hayes JM. **2002**. Practice and principles of isotopic measurements in organic geochemistry. [WWW document] URL https://www.whoi.edu/fileserver.do?id=73290&pt=2&p=74886. [accessed 29 July 2021].

Hermes JD, Roeske CA, O'Leary MH, Cleland WW. **1982**. Use of multiple isotope effects to determine enzyme mechanisms and intrinsic isotope effects. Malic enzyme and glucose 6-phosphate dehydrogenase. *Biochemistry* **21**: 5106–5114.

Laisk A, Edwards GE. **1997**. CO_2 and temperature-dependent induction in C_4 photosynthesis: an approach to the hierarchy of rate-limiting processes. *Functional Plant Biology* **24**: 505–516.

Melander LCS, Saunders WH. **1980**. *Reaction rates of isotopic molecules*. New York: John Wiley & Sons.

Noltmann EA. **1972**. 9 Aldose-ketose isomerases. In: Boyer PD, ed. The Enzymes. Academic Press, 271–354.

Rose IA, O'Connell EL. **1961**. Intramolecular hydrogen transfer in the phosphoglucose isomerase reaction. *The Journal of Biological Chemistry* **236**: 3086–3092.

Smith AM, Stitt M. **2007**. Coordination of carbon supply and plant growth. *Plant, Cell & Environment* **30**: 1126–1149.
